# Zika Virus: Promoting Male Involvement in the Health of Women and Families

**DOI:** 10.1371/journal.pntd.0005127

**Published:** 2016-12-29

**Authors:** Pauline E. Osamor, Christine Grady

**Affiliations:** Department of Bioethics, Clinical Center, National Institutes of Health, Bethesda, MD, United States; University of California San Francisco, UNITED STATES

Recent heightened media and public health attention to Zika virus (ZIKV) infection has focused on mosquito control, risks to pregnant women, and controversy over the summer Olympics. Missing from these messages is an emphasis on the essential role of men in decisions and behaviors related to ZIKV transmission and outcomes. It is our thesis that the role of men encompasses more than strategies to reduce risk of sexual transmission. Men play essential roles in decision-making affecting the couple and the family. The role of men is even more important in non-Western countries or patriarchal structures where husbands or other family members often control health-related decisions that are often considered the exclusive province of women in Western societies.

ZIKV, a flavivirus transmitted primarily by *Aedes* mosquitoes, has been spreading rapidly across the Western Hemisphere, where more than 60 countries and territories reported autochthonous transmission. In addition to mosquito-borne transmission, nonvector transmission routes, especially sexual ones, have also been described, but their impact during an epidemic and in nonepidemic settings remains largely unknown [1]. Most infections are asymptomatic, however people with ZIKV disease can have symptoms that include mild fever, skin rash, conjunctivitis, muscle and joint pain, malaise or headache, and certain less common serious neurological sequela [2]. Of particular concern is the association between ZIKV infection and adverse pregnancy and fetal outcomes including microcephaly.

## Sexual Transmission of ZIKV

The recognition of sexual transmission of ZIKV has had the consequence of a response from the reproductive health perspective. Sexually transmitted ZIKV infections were documented from symptomatic males who had traveled to regions where ZIKV was circulating [3,4]. In one instance, sexual intercourse occurred before the onset of symptoms, whereas in other cases sexual intercourse occurred during the development of symptoms or shortly after [5]. The risk factors and the duration of the risk of sexual transmission have not been determined. ZIKV has been detected by Polymerase Chain Reaction (PCR) in semen as long as 62 days after the onset of symptoms [6], demonstrating that a long period of infectivity might occur in nonvector transmission routes. However, it is not known if men infected with ZIKV who never develop symptoms can have the virus in their semen or spread ZIKV through sex. The observation that ZIKV can be sexually transmitted implies that mosquito-borne and sexual modes of transmission could bridge different groups of people and amplify transmission.

## WHO and CDC Guidelines on the Role of Men in ZIKV Infection

As a preventive measure, the World Health Organization (WHO) recommends that all people who have been infected with ZIKV and their sexual partners practice safer sex by using condoms correctly and consistently. Men who live in or have traveled to an area with ZIKV and whose partners are pregnant should practice safer sex, wearing condoms or abstaining throughout the pregnancy [7]. The US Centers for Disease Control and Prevention (CDC) suggests that both men and women who have had possible exposure to ZIKV via recent travel or unprotected sex with a ZIK-infected man wait for a period of time before trying to get pregnant [8]. The latest WHO interim guideline on prevention of sexual transmission of ZIKV provides more detailed guidelines for all people (male and female) [9]. Therefore, it appears that the total sum of the expected role of men in the control of ZIKV is limited to prevention of sexually transmitted infections (STIs). This limited role for men is inconsistent with what is known about the essential role played by men in women and children’s health and the known detrimental effects of absent male involvement on reproductive health.

## Role of Men in Reproductive Health Care

Historically, most reproductive health programs focused on family planning and, in turn, most family planning programs offer their services exclusively to women. Since contraceptives are mainly designed for women, women are targeted by family planning programs. In addition, reproductive health research, as well as policy and program formulation, has generally relied on data collected from women. However, even for fertility control and STIs, the inadequacy of strategies that target only women is well known [10]. Because of unequal gender–power relations, women are especially vulnerable but are often unable to negotiate changes in sexual behavior or to practice safe sex without the cooperation of their sexual partners.

The male role in reproductive health can span several themes. For example, men can be sources of transmission of STIs to women, often across networks of relationships. When women get pregnant, their partners are expected to be sources of financial, emotional, and other forms of support. They also participate in decision-making in matters affecting the pregnancy such as seeking health care and place of delivery. When pregnant women suffer complications, their partners are expected to assist with decision-making, payment for treatment, care of other children (if any), and provision of additional finances for the household if the woman is unable to work. Men as fathers play an important role in supporting their children from birth onwards. When a newborn child is affected by a disorder or is ill (including from maternal transmission of some STIs such as HIV or syphilis), the role of the father becomes even more critical, because the woman may find it difficult to cope with the multiple burdens of recovering from pregnancy and child birth, taking care of a sick child, making decisions about treatment, and planning for the likelihood of long-term care of a disabled child. Although women bear children, child rearing has an impact on men's lives too. This impact is substantial if men accept the responsibility of supporting their children in a range of other ways, including through ensuring the health and well-being of their partners and children [10].

An example of the call for an increased male role is the 1994 International Conference on Population and Development Program of Action’s statement on "Male Responsibilities and Participation" [11]: "Special efforts should be made to emphasize men's shared responsibility and promote their active involvement in responsible parenthood, sexual and reproductive behavior, including family planning; prenatal, maternal and child health; prevention of STDs, including HIV and prevention of unwanted and high-risk pregnancies.” It seems reasonable that if men are brought into a wide range of reproductive health services in such a way that they are supported as equal partners and responsible parents, as well as clients in their own right, better outcomes will be observed among both women and men. This will be reflected in reproductive health indicators such as contraception acceptance and continuation, safer sexual behaviors, use of reproductive health services, and reduction in reproductive morbidity and mortality.

## The Potential Role of Men in ZIKV Outbreak

For the control of the current ZIKV outbreak, it is essential to consider the many ways in which men should be involved. These include: decision-making about and practice of safer sex; negotiating sex while respecting women’s rights and decisions; decision-making about contraception and family planning; roles as husbands or partners of pregnant women who are at risk of being infected with ZIKV or are already infected with ZIKV; roles as fathers of Zika-infected children born with Zika sequelae; participation in research, including studies of natural history, infectivity, and clinical trials of vaccines and antiviral drugs; as well as support and advocacy of ZIKV control initiatives. These roles go well beyond current guidelines on safer sex, wearing condoms, and postponing conception, all of which are issues in which women alone rarely have the final say. We note that many of these issues also hold true for other situations as varied as the HIV/AIDS pandemic and Ebola virus disease. In other words, situations in which a viral infectious agent which causes severe morbidity and/or mortality and can infect all age groups can be sexually transmitted and which requires a multipronged approach to control.

While highly desirable, expanded male participation in efforts to reduce sexual transmission of ZIKV faces a number of potential barriers. These include the fact that male dominant decision-making processes are prevalent in many low and middle-income countries (LMIC). Decisions about whether and when to seek health care and use of contraceptives are made by men. This is one of the major causes of gender inequality in reproductive health. Another barrier is the perception that contraception, family planning, pregnancy, and childbirth are seen as belonging to the domain of women’s health. While this stems from social norms that assign the role of childbearing and child rearing to women, this perspective often carries over to health care systems in many communities. Without a change in this perspective, men can be seen as interlopers when attempting to involve themselves in reproductive health systems. A further complexity is the fact that social, tribal, and/or religious norms may prohibit or discourage the open discussion of sexual matters. This often means that couples feel uncomfortable talking about their family planning needs and sexual concerns with their partners and with health educators. Most of these barriers are sociocultural and are often society-specific [12, 13]. However, experience with the HIV/AIDS pandemic has facilitated the development of ways to mitigate these problems [14]. Such lessons are useful in the control of ZIKV.

## Recommendations/Suggestions

Recognition of these potential roles for men and how to incorporate them into control efforts could bring a more effective community response to the outbreak. Equally important is that it will frame the ZIKV outbreak as a community problem, not just a woman’s problem.

While acknowledging that circumstances vary by country and other contexts (e.g., rural versus urban settings), one approach is to utilize the existing modes of communicating health information in the communities. These include use of the electronic and print mass media, health education in community health centers, and specific health education campaigns. It is often useful to enlist the aid of leadership groups, such as village elders, church leaders/ministers, and leaders of men’s cultural groups to get men involved.

Key ways men can be directly involved in women’s reproductive health include:

1. Tailoring male-inclusive interventions to the specific society/sociocultural contexts. In cultures where men are the primary decision makers in most matters (including fertility and family planning), the best ways to communicate with them and involve them may not be through their spouses. Local public health professionals and community leaders are often the best channels of introducing such interventions.
2. Provide health education in other settings. Health education should go beyond when partners accompany their partners for antenatal clinics (ANCs), which is not always the case due to cultural barriers.
3. Involve men in all matters that require joint spousal decisions, as this is crucial to achieving good reproductive health goals and care of children, especially in patriarchal societies.
4. Involve men in counseling sessions. This can help make them more supportive of contraceptive use and more aware of the concept of shared decision-making.
5. Involve men in program design and implementation. Men themselves are the best sources of information about effective outreach and service delivery strategies. As such, it is essential to involve them throughout program design and implementation to ensure that services and informational materials address their concerns and needs. Many programs also use men to implement the program as staff members, health educators, and peer motivators.
6. It is also recommended that testing of both partners be done during ANC visits the same way HIV testing is currently done.

Therefore, we recommend that men be involved in as many components of ZIKV control efforts as possible. The health of their spouses, unborn children, sisters, mothers, and other family members is at stake. Public health education efforts should help men be aware of and follow public health guidelines during a ZIKV outbreak, highlighting the fact that minimizing risk of ZIKV transmission benefits not only themselves but also their families. These efforts would help men be fuller partners with their spouses, especially in the countries where ZIKV outbreaks are occurring.

## Disclaimer

The opinions expressed are the authors’ and do not necessarily reflect the positions or policies of the National Institutes of Health or the US Department of Health and Human Services.
